# Exome Sequencing Reveals Immune Genes as Susceptibility Modifiers in Individuals with α_1_-Antitrypsin Deficiency

**DOI:** 10.1038/s41598-019-49409-1

**Published:** 2019-09-11

**Authors:** Chiara Rigobello, Simonetta Baraldo, Mariaenrica Tinè, Ilaria Ferrarotti, Angelo Guido Corsico, Erica Bazzan, Graziella Turato, Elisabetta Balestro, Davide Biondini, Giorgio Valle, Marina Saetta, Manuel G. Cosio

**Affiliations:** 1 Department of Cardiac, Thoracic, Vascular Sciences and Public Health, University of Padova, Padova, Italy; 2 Center for Diagnosis of Inherited Alpha 1-antitrypsin Deficiency, Department of Internal Medicine and Therapeutics, University of Pavia, Pavia, Italy; 3 Division of Respiratory Diseases, IRCCS Policlinico San Matteo Foundation, Pavia, Italy; 4 CRIBI Biotechnology Center, University of Padova, Padova, Italy; 5 Meakins-Christie Laboratories, Respiratory Division, McGill University, Montreal, Canada

**Keywords:** Genome, Sequencing

## Abstract

Alpha-1 antitrypsin deficiency (AATD) is a genetic disorder associated to early onset emphysema, mainly imputable to Pi*ZZ genotype. In spite of the serious potential effects, many AATD individuals do not develop emphysema. To identify genes/variants potentially involved in emphysema development we studied 4 AATD families. Each family had at least one affected sibling with emphysema and one non-affected. Whole Exome Sequencing (WES) was performed on genomic DNA isolated from 9 individuals with AATD (4 affected/5 non-affected). Genetic variants confirmed at least in three families were prioritized using QueryOR and network analysis was used to verify enriched pathways. In affected subjects: 14 genes (57% immune-related) segregated in a recessive model and 21 (29% immune-related) in a dominant model. In non-affected subjects: 21 genes (43% immune-related) segregated in a recessive model and 50 (24% immune-related) in a dominant model. In affected siblings immune genes had an activating function, while where immune-suppressing in non-affected siblings involving antigen processing, MHC-I presentation, TCR and PD-1 signalling. This study describes possible genetic susceptibility factors for emphysema development in AATD, and suggests that gene variants involved in regulation of immune homeostasis and maintenance of self-tolerance contribute to the development or suppression of the disease.

## Introduction

SERPINA1 gene codifies for α-1 antitrypsin (AAT), a protease inhibitor, archetype member of the superfamily of Serine Protease Inhibitors (SERPINs) characterized by their ability to inhibit proteases that possess the amino acid serine in their active site. AAT is part of a serpins cluster on chromosome 14q32.1 that includes: corticosteroid binding globulin (SERPINA6), α-1 antitrypsin-like pseudogene, α-1 antitrypsin (SERPINA1), protein C inhibitor, α-1 chymotrypsin. This group of genes is in conserved linkage with the immunoglobulin heavy chain gene^1^. AAT is the main inhibitor of proteases in the lung, providing more than 90% of the defence against the elastolytic activity of neutrophil elastase^2^. As an acute phase reactant, its tissue concentrations can increase by as much as 11 folds during an inflammatory response and, as recently become evident, AAT has important regulatory and anti-inflammatory properties, affecting both the innate and adaptive immune systems^3–5^.

The wild type form of SERPINA1, corresponding to the “M” allele (PI*M), produces normal levels of serum protein. Most of the severe α_1_-antitrypsin deficiency (AATD) cases are associated with PI*ZZ genotype, a result of a missense mutation (p.E366K-c.1096G > A rs28929474) causing an aminoacidic substitution from a negatively charged Glutamate (GAG) to a positively charged Lysine (AAG). This mutation induces a conformational change favouring abnormal protein folding and polymerization, resulting in AAT retention within the endoplasmic reticulum of the liver and, consequently, very low serum AAT levels. Some very rare variants of the SERPINA1 are secondary to insertion/deletion, frame-shift, stop codon mutations, with a consequent lack of production of the AAT protein and no detectable plasma levels (PiNull)^6,7^.

AATD is one of the most common respiratory hereditary disorders affecting Caucasians of European descent^8^. The genetic abnormality is inherited in an autosomal co-dominant pattern and is characterized by a high risk for development of severe emphysema, often at early age, and a lesser risk for the development of liver disease and, rarely, panniculitis^8,9^. The pathogenesis of emphysema in this condition is complicated and involves not only the loss of the antielastase properties of AAT, but also a severe adaptive immune inflammation in the lung, as it has been recently shown^3^. The inflammatory immune reaction is not surprising in view of the potent anti-inflammatory properties of AAT, which are lost with its deficiency^3,10^.

In spite of its serious potential effects, the development of emphysema in the setting of severe AAT deficiency is highly variable and there is a large discrepancy between the predicted numbers of PI*ZZ subjects in the population and the actual number of individuals that are recognized to have lung disease^11–13^. Furthermore, even when cigarette smoking (an important risk factor for the development of emphysema in PI*ZZ individuals) is considered, there is a substantial variability on the severity of pulmonary disease: lung function can be well preserved in some PI*ZZ smokers, but severely impaired in some non-smokers^14^. Such evidences strongly suggest that the phenotypic expression of the disease could be influenced by an incomplete penetrance and/or variable expressivity of the PI* Z allele. This finding is not surprising since it has become evident that traits inherited in a simple Mendelian fashion are rare in nature, due to the interaction with susceptibility genetic modifiers and environmental factors, which could affect phenotypes in subtle or profound ways^15,16^. Likely, the presence of genetic susceptibility modifiers could explain the variable phenotype seen in AATD.

In this study we used Whole Exome Sequencing (WES) technology^17^ to identify variants that might potentially account for the variability in penetrance and expressivity of the disease induced by the AATD genetic disorder. Towards this aim, we analysed whole exome in AATD siblings within the same family with extreme phenotypes of the disease (one sibling affected and one non-affected), and compared the variants across unrelated families. Restricting the analyses to AATD siblings with opposite phenotypes could limit heterogeneity and help the identification of factors contributing to the development of emphysema^18^. Some of the results of this study have been reported in the form of an abstract^19^.

## Results

### Study population

We studied 4 families from the AATD Italian Registry with siblings concordant for genotype, but with discordant phenotypes (emphysema/no emphysema). Clinical characteristics are reported in Table 1. All siblings had severe AATD, defined by the carriage of the PI*ZZ (families 3, 185, 237) or PI*Z/Q0_brescia_ genotypes (family 114). Whole Exome Sequencing confirmed the genotype in all individuals. Each family studied had at least one affected sibling with emphysema and a non-affected sibling, free of disease. The population studied comprised 9 subjects, 4 affected and 5 non-affected, with a predominance of females (n = 6; 66.7%) and a mean age of 56.5 ± 15.5 years old. One of the affected subjects had normal FEV_1_ but had significant dyspnea, emphysema and bronchiectasis by CT scan and reduced diffusion capacity (Table 1). Two of the affected subjects were ex-smokers with a smoking history of less than 20 pack-years and who had quit smoking for more than 10 years; the rest were never smokers.

**Table 1:** Subjects characteristics.

Family ID	Subjects ID	Sex	Age (yrs)	AAT level (mg/dL)	Genotype	FEV_1_ (% pred)	Smoking History
3	AWI	F	67	18	ZZ	94	Non Smoker
	AWJ	M	65	43	ZZ	106	Non Smoker
	AWK*	**M**	**60**	**22**	**ZZ**	**25**	**Ex Smoker**
185	**AWL**	**F**	**73**	**37**	**ZZ**	**57**	**Non Smoker**
	AWM	F	71	36	ZZ	85	Non smoker
237	**BIR**	**F**	**53**	**28**	**ZZ**	**102** ^**†**^	**Non Smoker**
	BQ0	F	43	35	ZZ	100	Non Smoker
114	**BIP**	**M**	**50**	**23.3**	**Z/Q0**	**32**	**Ex Smoker**
	BIQ	F	47	20	Z/Q0	99	Non Smoker

Bold characters identify affected siblings.

*This patient underwent lung transplantation.

^†^In spite of preserved FEV_1_, this patient suffered from dyspnea and productive cough. The diffusing capacity of the lungs for carbon monoxide (DL_CO_) was reduced and thoracic HRCT showed emphysema and bronchiectasis.

### WES analysis

Whole Exome Sequencing generated an average of 3.6 × 10^7^ reads mapped to the reference genome at a mean depth of 105.4-fold coverage per sample. The 93.5% of exome was covered at minimum with 20X, and a uniformity >91%. Base calls with Phred Quality Score <20 (Q20) were considered low quality and discarded. All the examined parameters (mapped reads, reads on target, mapped bases, average coverage, uniformity, transition/transversion ratio) exceeded the normal standards applied for the identification of germline variants, making our results robust (a complete description of WES statistic, coverage and type of variants is shown on Supplementary Tables S1, S2 and Supplementary Fig. S1). After quality control adjustment, a total of 77204 variants remained for analysis. Table 2 summarizes the type of variants found in our population. For all subsequent analysis, we excluded variants located on not confirmed transcripts (20730) or on Untranslated Regions (5′UTR and 3′UTR) (14597). Using ANNOVAR and dbNSFP database (v.2.9), 41877 functionally annotated variants were identified: 49.5% (20748) synonymous and 50.5% (21129) non-synonymous. Among the non-synonymous 19751 were missense, 240 nonsense, 612 frameshift, 447 inframe, 59 stop-loss and 20 stop-gain. Of the 77204, 7.6% (5914) variants were classified as rare variants (GMAF ≤ 1%), 4.3% (3345) were novel and 1.4% (1058) were insertions-deletions.

**Table 2:** Genetic Variants Identified in Population Using WES.

Genetic Variants Identified in Population Using WES				
Subjects				9
All variants				77204
	*Not confirmed transcript*			20730
	*UTRs*			14597
	Functionally annotated			41877
		Synonymous		20748
		Non-synonymous		21129
			Missense	19751
			Nonsense	240
			Frame-shit	612
			In-frame	447
			Stop-loss	59
			Stop-gain	20
**Rare**				
(GMAF < 1%)				5914
Novel				3345
Deletion				645
Insertion				413

Definition of abbreviations: GMAF = global minor allele frequency.

### Variant filtering and annotation

**Figure 1:**
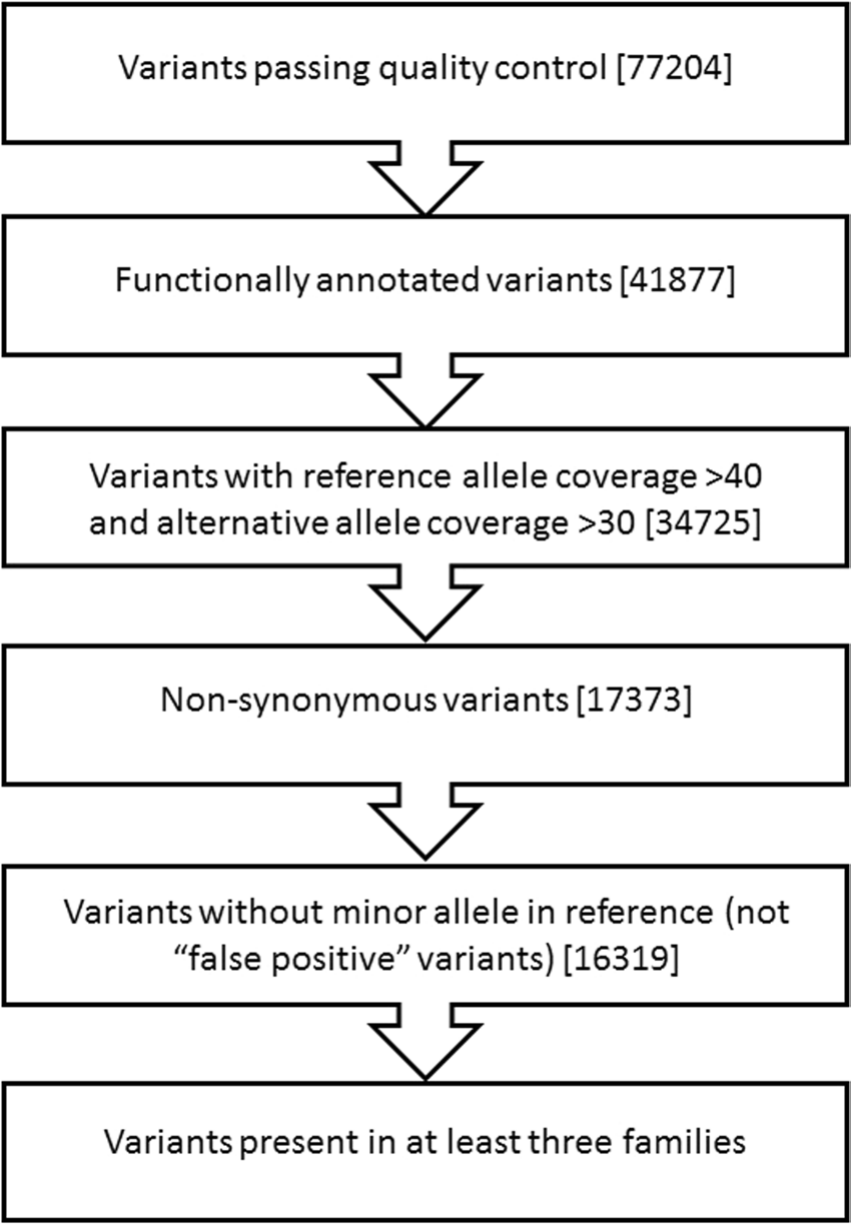
Flowchart of variant filtering process. Variants identified were filtered as described in methods. The number of remaining variants after each step is shown in square brackets.

A flow chart of the filtering approach applied to the 77204 variants found is shown in Fig. 1. Among the 41877 functionally annotated variants (synonymous and non-synonymous), we considered only high confidence variants with alternative allele coverage >30 (34725). After synonymous exclusion, non-synonymous variants were 17373. In addition, after excluding variants with minor allele in reference (false positive)^20^, the number of variants was 16319.

**Figure 2:**
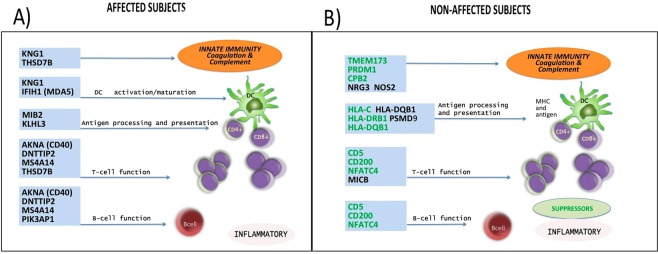
The discriminating genes with immune functions and their targets. Panel A shows variants with recognized immune function in the affected subjects. All these genes have been described to exert a pro-inflammatory function (pro-inflammatory genes in black font). Panel B shows variants with recognized immune function identified in the non-affected subjects. Most of these genes exert an immune suppressor function (suppressor genes in green font, pro-inflammatory genes in black font).

As the number of potential segregating variants is large, particularly in small family units (sibling pairs), we have limited the number of candidate genes restricting variants analysis to those confirmed at least in three families and we have taken into consideration two patterns of inheritance: recessive and dominant models. Table 3 shows variants identified in affected subjects: 14 genes with 15 variants in the recessive model (57% immune-related) and 21 genes with 23 variants in the dominant model (29% immune-related). Figure 2A shows genes with recognized immune function identified in the affected subjects, none of which were present in the non-affected subjects. All these genes have been described to exert a pro-inflammatory function, affecting key steps involved in the immune response, such as: coagulation, complement and innate immunity (KNG1, THSD7B, IFIH1/MDA5), DC activation/maturation (IFIH1/MDA5, KNG1), ubiquitination antigen processing and presentation (MIB2, KLHL3), T cell function (AKNA, MS4A14, THSD7B, DNTTIP2) and B cell function (AKNA, DNTTIP2, MS4A14, PIK3AP1).

**Table 3:** Variants identified in affected subjects.

Gene name	SNP ID	wild type allele	AWK (family 3)	AWL (family 185)	BIR (family 237)	BIP (family 114)
**Recessive Inheritance Model**						
**AKNA**	rs3748176	G		A/A	A/A	A/A
**DNTTIP2**	rs3747965	C		A/A	A/A	A/A
FRMD1	rs1548349	G	C/C	C/C		C/C
HJURP	rs3806589	T		C/C		C/C
HJURP	rs3732215	G	C/C	C/C		C/C
**IFIH1**	rs3747517	T		C/C	C/C	C/C
**KLHL3**	rs2905608	T	C/C	C/C		C/C
**KNG1**	rs710446	T	C/C	C/C	C/C	
**MIB2**	rs7418389	T		C/C	C/C	C/C
**MS4A14**	rs3217518	ATT		A/A	A/A	A/A
RTP2	rs11707167	T	C/C		C/C	C/C
SLC22A16	rs714368	T	C/C	C/C		C/C
**THSD7B**	rs10206850	A	G/G	G/G	G/G	
TMPRSS5	rs7110736	A		G/G	G/G	G/G
TSPAN8	rs3763978	C		G/G	G/G	G/G
**Dominant Inheritance Model**						
ACBD3	novel*	CTTTTTTTT	CTTTTTTT	CTTTTTTT		CTTTTTTT
**ALAD**	rs1800435	C		C/G	C/G	C/G
**APEX1**	rs1130409	T		T/G	T/G	T/G
EGFL8	rs3096697	G		G/A	G/A	G/A
HSD17B4	rs25640	G	G/A	G/A	G/A	
IQCG	rs9880989	G	G/T		G/T	G/T
**KLC2**	rs2276036	C	C/T		C/T	C/T
L3MBTL4	rs3737353	C		C/T	C/T	C/T
LIPK	rs1214464	G	G/C	G/C	G/C	
LRCH4	novel*	GTC		GC	GC	GC
MKI67	rs8473	T	T/C	T/C	T/C	
MKI67	rs11106	G	G/C	G/C	G/C	
MS4A12	rs2298553	C	C/T	C/T		C/T
PCDH12	rs164515	C	C/T	C/T		C/T
**PIK3AP1**	rs17112076	C		C/T	C/T	C/T
**PPT2**	rs3096696 (EVS)	C		C/A	C/A	C/A
**PRDM16**	rs2493292	C		C/T	C/T	T/T
TBC1D26	rs11650318	C	C/T		C/T	C/T
TBC1D26	rs17855672	G	G/A		G/A	G/A
TRIM16	rs1060903	C		C/A	C/A	C/A
TSPYL1	rs3828743	G	G/A		G/A	G/A
VSTM4	rs13088	A		A/G	A/G	A/G
ZNF286A	rs3760299	T	T/C		T/C	T/C

Definition of abbreviations: SNP: single nucleotide polymorphism; EVS = present in Exome Variant Server. *novel = not present in any public database. Genes linked to immunity are shown in bold and underlined characters.

57% of the genes in the recessive inherited model and 29% in the dominant were immune.

Table 4 shows variants identified in non-affected subjects: 21 genes with 21 variants in the recessive model (43% were immune-related genes), and 50 genes with 62 variants in the dominant model (24% were immune-related genes). Figure 2B shows genes with a recognized immune function identified in the non-affected subjects, none of which was present in the affected subjects. Contrary to what was found in the affected subjects, a significant number of the gene expressed in the non-affected group have been described to exert a suppressor function at the different steps of the immune cascade: complement and innate immune response (CPB2, PRDM1, TMEM173), antigen presentation (HLA alleles, mainly DRB1 DQB1), T and B cell functions (CD5, CD200, NFATC4).

**Table 4:** Variants identified in non-affected subjects.

Gene name	SNP ID	wild type allele	AWK (family 3)	AWL (family 185)	BIR (family 237)	BIP (family 114)
**Recessive Inheritance Model**						
AAK1	rs66931661	CTGT	C/C		C/C	C/C
ABCB11	rs2287622	A	G/G	G/G		G/G
ALG1L	rs3828357	T	C/C	C/C	C/C	
C1orf227	rs10864004	A	G/G		G/G	G/G
**CD5**	rs2229177	C	T/T	T/T		T/T
**CPB2**	rs1926447	A	G/G		G/G	G/G
CSNK1A1L	rs9576175	G	T/T		T/T	T/T
**HLA-C**	rs79636386	A		C/C	C/C	C/C
**HLA-DQB1**	rs1049130	A	G/G	G/G	G/G	
KRTAP19-4	rs2298437	T	C/C	C/C		C/C
LCE5A	rs2105117	G	A/A	A/A	A/A	
MMRN2	rs3750823	C	T/T	T/T		T/T
**NFATC4**	rs7149586	T	C/C		C/C	C/C
PM20D1	rs1361754	A	G/G	G/G		G/G
**RTP1**	rs6764714	C	G/G		G/G	G/G
**SIM2**	rs2073601	C	A/A	A/A	A/A	
TINAG	rs1058768	T	C/C	C/C	C/C	
TMED5	rs1060622	G		A/A	A/A	A/A
**TMEM173**	rs1131769	T	C/C		C/C	C/C
TPTE	novel*	G		T/T	T/T	T/T
**ZC3H13**	rs9534264	T	A/A		A/A	A/A
**Dominant Inheritance Model**						
ACBD3	rs2306120	T	T/G	T/G		T/G
ALPK2	rs9944810	C		C/A	C/A	C/A
ANKK1	rs1800497	G	G/A	G/A		G/A
ART4	rs11276	C	C/T	C/T		C/T
BBS12	rs309370	G		G/A	G/A	G/A
BBS12	rs13135778	G		G/A	G/A	G/A
C12orf60	rs139293175	CTA	CTA/C	CTA/C		CTA/C
C12orf60	rs7307438	T	T/A	T/A		T/A
CCDC144NL	rs79930314	G	G/T		G/T	G/T
CCDC144NL	rs79843086	G	G/T		G/T	G/T
CCHCR1	rs130068	G	G/A	G/A	G/A	
**CD200**	rs2272022	C	C/A	C/A	C/A	
CWF19L2	rs659040	G	G/A		G/A	G/A
CYP21A2	rs397515530	G		A/A	A/A	G/A
DHRS4	rs17099455	G		G/A	G/A	G/A
DYNC2LI1	rs9309107	T	T/A	T/A	T/A	
EDN1	rs5370	G	G/T		T/T	G/T
EFS	rs2231798	T	T/C	T/C	T/C	
EPCAM	rs1126497	T	T/C		T/C	T/C
FHAD1	rs4661330	A	A/G	A/G		A/G
HINFP	rs100803	C	C/T	C/T		C/T
**HLA-C**	rs79636386	A	A/C	C/C	C/C	C/C
**HLA-DQB1**	rs1130398	C	C/T	C/T	C/T	
**HLA-DQB1**	rs1063323	C	C/T	C/T	C/T	
**HLA-DRB1**	rs71547382	C		C/T	C/T	C/T
KIF20B	rs1886997	A	A/G	A/G		A/G
KIF20B	rs144593231	C	C/CTAAAAG	C/CTAAAAG		C/CTAAAAG
KLHL33	rs1953225	T	T/C		T/C	T/C
KRT40	rs9908304	G	G/A	G/A		G/A
LRRC6	rs2293979	G	A/A	G/A		G/A
**MCCD1**	rs2259435	G	G/A	G/A	G/A	
**MICB**	rs1065075 (EVS)	A	A/G	A/G	A/G	
**MICB**	rs1051788 (EVS)	G	G/A	G/A	G/A	
MMP27	rs1276286	T	T/A	T/A	A/A	
**NOS2**	rs2297518	G	G/A	G/A		G/A
NPAS2	rs9223	C	C/T	C/T	C/T	C/T
**NPY4R**	rs79871698	G	G/A		G/A	G/A
**NRG3**	rs1884282	C	C/G	C/G		C/G
NSUN4	rs3737744	A	A/G	A/G	A/G	
**NUMBL**	rs749669311	T	TTGCTGTTGCTGCTGCTGC/T	TTGCTGTTGCTGCTGCTGC/T	TTGCTGTTGCTGCTGCTGC/T	TTGCTGTTGCTGCTGCTGC/T
OR10G2	rs41314525	C	C/A	C/A		C/A
OR13C2	rs10156474	T	T/C		T/C	T/C
OR13C2	rs10991326	A	A/T		A/T	A/T
OR13C5	rs4117966	C	C/T		C/T	C/T
OR13C5	rs1523678	T	T/C		T/C	T/C
OR13C5	rs1851725	A	A/G		A/G	A/G
OR13C5	rs6479260	G	G/C		G/C	G/C
OR13C5	rs11314210	GT	GT/G		GT/G	GT/G
OR13C9	rs993658	T	T/A		T/A	T/A
OR2T2	rs67700848	C	C/T	C/T		C/T
OR4E2	rs61732411	C	C/T	C/T	C/T	C/T
**PRDM1**	rs811925	C	C/G	C/G		C/G
**PSMD9**	rs14259	A	A/G	A/G		A/G
RNLS	rs2296545	C	C/G	C/G		C/G
SPATA16	rs1515441	C		C/T	C/T	C/T
SPATA16	rs16846616	C		C/T	C/T	C/T
SVEP1	rs3739451	A	A/T		A/T	A/T
TMEM132C	rs12424159	G	G/A	G/A	G/A	
TMEM71	rs1895807	A	A/G	A/G		A/G
WDR66	rs17852561	C	C/T	C/T		C/T
WWC2	rs11734376	G	G/T		G/T	G/T
ZNF626	rs73002662	T	T/G	T/G		T/G

Definition of abbreviations: SNP: single nucleotide polymorphism; EVS = present in Exome Variant Server. Genes linked to immunity are shown in bold and underlined characters. *novel = not present in any public database.

43% of the genes in the recessive inherited model and 24% in the dominant were immune.

None of the 5914 rare variants (GMAF ≤ 1%) were confirmed in three families, using our filtering criteria. Three novel variants were identified: TPTE 21:g.10951387T > G, missense, (in non-affected subjects, recessive inheritance model); ACBD3 1:g.226352490CTTTTTTTT > CTTTTTTT, frameshift and LRCH4 7:g.100175472GTC > GC, frameshift (in affected subjects, dominant inheritance model). The alignment of these variants against the eutherian mammals in the Ensembl database show that they were all located in not-conserved regions suggesting that these substitutions were of little significance.

The implication of amino acids changes on protein function in affected and non-affected subjects under the two inheritance models according to CADD, DANN and PROVEAN scores rating are shown in the Supplementary Tables S3–S6.

### Pathways analysis

**Figure 3:**
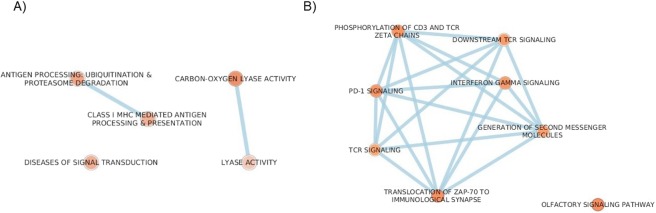
Enrichment maps in Cytoscape3. This analysis integrates and visualizes data from Gene Ontology, KEGG and Reactome. Panel A shows the networks in affected subjects; panel B in non-affected subjects. The intensity of the colour in the node corresponds to level of significance (the darkest the node, the most significant the p-value).

Using Reactome and KEGG we investigated the possible functional significance, network interactions and biological pathways of the whole number of genetic variants, differentially found in affected or non-affected individuals. Pathways mainly related to the immune system were significantly represented both in affected and non-affected subjects using Reactome (Supplementary Tables S7–S10) and confirmed in KEGG (data not shown). The pathways enriched in non-affected subjects such as cytokine signaling in immune system, interferon signaling, class I MHC mediated antigen processing and presentation were significant, even after False Discovery Rate (FDR) correction (Supplementary Tables S8, S10). Finally, enriched pathways were integrated using Enrichment map app in Cytoscape3, which translates data to a network where highly similar terms in both databases cluster together. This network analysis is shown in Fig. 3. Panel A shows the network analysis in affected subjects: enriched pathways include those related to the immune system (antigen processing and class I MHC antigen presentation). Other minor networks included diseases of signal transduction and lyase activity, possibly linked to DNA repair in response to oxidative stress. Panel B shows the network analysis for non-affected subjects: pathways of immune system (Interferon Gamma, TCR and PD-1 signalling) are predominantly enriched.

## Discussion

The genetically induced α-1 antitrypsin deficiency (AATD) predisposes to the development of emphysema in which a severe adaptive inflammatory reaction, similar to the one seen in COPD with normal AAT levels, plays an important role^3,21,22^. However, as seen in other genetically induced abnormalities, individuals with AATD often fail to express most, if not all, features of disease (emphysema, COPD), possibly due to the action of modifier genes. It is thus likely that the variable penetrance and expressivity of the Pi*ZZ genotype may reflect the action of unlinked modifier variants, a possibility that has never been investigated.

In this work, we used Whole Exome Sequencing (WES) to identify potential modifier genes/variants that might contribute to the development of emphysema in AATD. Our study of siblings of opposed phenotypes in four unrelated families, has provided description of sets of variants shared exclusively by affected subjects or exclusively by non-affected subjects with AATD. These results highlight the likely involvement of immunity in the development or suppression of the disease in subjects with AATD.

To our knowledge, this is the first description of possible genetic susceptibility factors influencing the risk of developing or avoiding the lung disease in AATD and possibly, by extension, in COPD with normal AAT levels. At difference with previous reports using WES in COPD, we did not filter for the exclusion of common variants since it is known that the phenotypic effects of some rare alleles are modified by common alleles^15,16^. The variants segregating in affected and non-affected subjects differed substantially between the two groups, suggesting that different genetic factors might be modulating the penetrance of the AATD gene and thus the phenotype.

In the affected siblings, 14 genes with 15 variants in the recessive model (homozygosity), and 21 genes with 23 variants in the dominant model (heterozygosity) were identified in at least three out of four different pedigrees. Fifty-seven percent of the variants in the recessive model and 29% in the dominant model have recognized important roles in innate and adaptive immunity, affecting activation of complement cascade, antigen presentation and regulation of immune responses as shown in Fig. 2. Of particular interest among the genes found, is the presence in the affected pedigrees of the rs3747517 variant on IFIH1 gene that has been strongly associated with susceptibility to autoimmune diseases^23,24^. This gene codes for an innate immune protein, which induces the type I interferons and pro-inflammatory cytokine cascades. Similarly pertinent, because of their involvement in autoimmunity, are AKNA and MIB2 genes. AKNA codifies for a transcription factor that specifically activates the expression of CD40 receptor/ligand and is critical for antigen presentation, B and T cell development^25^. The MIB2 gene is an ubiquitin-protein ligase involved in the class I MHC mediated antigen processing and presentation^26^.

Importantly, some of the genes we detected in the affected subjects (PPT2, DNTTIP2, IQCG and PRDM16) have previously been reported in Genome Wide Association Studies (GWAS) or transcriptomic analyses on patients with usual COPD. PPT2 is a thioesterase multiligand receptor of the immunoglobulin superfamily, which has been associated with FEV_1_ values in the general population^27,28^ and has been implicated in the pathogenesis of COPD^29^ and in the progression of emphysema^30^. This gene has also been shown to be a risk locus for rheumatoid arthritis^31^. DNTTIP2 (a regulator of chromatin remodelling involved in B cells differentiation and maturation^32^, IQCG (a calcium and calmodulin-dependent protein kinase) and PRDM16 (a repressor of TGF-beta signalling) have been shown to have enriched gene expression in patients with COPD and/or emphysema^33,34^. The reproduction of these associations across different cohorts and different race/ethnic groups highlights the importance of variations in these genes in the mechanisms of COPD with and without AATD.

In the non-affected siblings, 21 genes with 21 variants in the recessive model, and 50 genes with 62 variants in the dominant model were identified and shared in at least three of the four families. At difference with the affected subjects a high proportion of these variants had a known immune suppressor function (Fig. 2B). Forty-three percent of the variants in the recessive model and 24% in the dominant model have recognized important roles in innate and adaptive immunity. Among these, variants in HLA-C, HLA-DQB1 and HLA-DRB1 genes were found, a very significant finding in view of the important contribution of the HLA complex to immune disease susceptibility and pathogenesis. On this line, the MHC class II molecules HLA-DQB1 and HLA-DRB1, along with the MCH class I HLA-C, have been recently associated with resistance to systemic autoimmune diseases, such as systemic sclerosis, systemic lupus erythematosus and rheumatoid arthritis^35,36^.

Possibly related to the lack of AATD induced abnormalities in non-affected subjects, is the considerable number of genes with a clear immune suppressive/regulatory function (Fig. 2B). CD5, a lymphocyte surface receptor, constitutively expressed by all T cells and a subset of mature B cells, is particularly important in the context of our study, since the variant we identified rs2229177 has been associated with better clinical outcomes in autoimmune diseases^37^. CD5, acting as an immune inhibitor checkpoint equally to PD-1 and CTLA-4, is key in the maintenance of immune homeostasis^38^.

The TMEM173 allele R232H, corresponding to our homozygous variant rs1131769, is a major regulator of the innate immunity and interferon response^39^. Furthermore, CD200 (a membrane glycoprotein belonging to the immunoglobulin superfamily), PRDM1 and CPB2 were the other genes with important immune suppressive functions found in the non-affected siblings, but not in affected ones^40^. Cytoscape network analysis confirmed that variants identified in our population have biological functions and important network interactions, which fit with the described immune mechanism in the production of emphysema^3,21,22^. Overall, our results point towards a large contribution of regulatory molecules involved in the normal homeostasis of immune responses and the maintenance of self-tolerance. These factors act synergistically to orchestrate an immunological environment which could be conducive and promote the development of emphysema or, what is more relevant, to confer a resistance status, evading the disease. These findings support the concept that activation, or failure of suppression, of the innate and adaptive immune response, may well be responsible for the development of COPD and emphysema, both in AATD^3^ and COPD with normal AAT levels^21–23^.

Our study has several limitations: the number of families studied is small, but it is an accepted norm in studies of Mendelian traits, especially when comparing opposed phenotypes in family settings^16,18^. Indeed, WES is a powerful method to investigate genetic variation even within a small number of individuals. In particular, in human diseases characterized by considerable heterogeneity, these new technologies overcome the analysis of a single polymorphism, allowing to consider the effect of a set of variants in their entirety. Despite the small size of our study we were able to replicate some of the findings of previous GWAS studies on lung function^27–30^ and co-expression network analyses in COPD^33,34^, expanding the evidence linking immune system and lung abnormalities^41–44^. The lack of quantitation of the extent of emphysema by computer-assisted analysis is a possible limitation in our study. However, all CTs were assessed independently by two thoracic radiologists and supported by measurements of diffusion capacity that can enhance the detection of emphysema^45^. Another limitation to be considered would be the potential bias of smoking. There were two individuals with a smoking history in the study and, although marginal (ex-smokers of less than 20 pack-years who had stopped smoking from more than 10 years), they had the lowest lung function of the group. Finally, although the findings in the study are suggestive of an immune component playing a role in the development or avoidance of the disease, there is a need for further validation studies to confirm the associations found.

In conclusion, to our knowledge, this is the first study that provides a detailed description of possible genetic susceptibility factors for emphysema in siblings with AATD. Our findings suggest that gene variants mainly involved in the regulation of immune homeostasis and the maintenance of self-tolerance could contribute to the development or suppression of the disease. The evidence that some of the genetic determinants identified in our study were previously described by GWAS in the development of COPD with normal AAT levels, adds to the significance of our results and highlights the similarities in the mechanisms of COPD/emphysema with or without AATD.

## Methods

Extended methods for each aspect of the study and analysis plan are provided in the Online Supplement. Briefly, the study included 9 individuals from 4 different families in the Italian Registry for AATD^46^. The probands selected from the registry were aged ≥18 years with the presence of severe AAT deficiency, defined by the carriage of the PI*ZZ genotype, or null genotypic variants. Within the same family, we compared siblings who were concordant for genotype, but discordant for clinical presentation (i.e. emphysema or no emphysema). The presence of emphysema in the affected sibling (and its absence in non-affected ones) was determined by chest HRCT. To confirm that non-affected subjects were free of any respiratory disease we also required them to have a preserved lung function with normal diffusing capacity for carbon monoxide (DL_CO_) and normal blood gases. To minimize the potential confounding effect of smoking, we have selected among the families in the Italian registry those whose smoking history was unremarkable (either nonsmokers or former smokers who had quit by >10 years and had <20 pack-years cumulative exposure). The study was approved by the Institutional Review Board (Policlinico San Matteo, Pavia) and complied with the principles set out in the Declaration of Helsinky. Informed consent was obtained from each participant regarding storage of biological samples and genetic sequencing. The clinical results are presented here in a fully anonymous form.

DNA was extracted from whole blood and genotyping performed as previously described^47^. Exome libraries were prepared using the Ion Proton Targeted Sequencing Library (Ion AmpliSeq™ Exome RDY Kit) with a minimum coverage of 80X. Data were processed with standard methods (see Supplementary Methods).

To filter and prioritize the identified variants, we used QueryOR^20^, a new online pipeline developed by the bioinformatics unit of our university. We included only high confidence variants and variants that were non-synonymous, and excluded false positive variants in the reference genome. We did not apply any further filtering criteria, including both novel and annotated variants.

We analyzed affected and non-affected subjects considering different inheritance models: (a) recessive model (*shared homozygosity* in QueryOR); (b) dominant model (*shared variants* in QueryOR). The analysis was restricted to variants confirmed at least in three families out of four.

ANNOVAR^48^ and dbNSFP database (v.2.9)^49^ were used to integrate annotation, linking variants to genes, transcripts, proteins and biological ontologies. To further estimate the impact of variants on protein structure and function, we used scores that consider the functional impact on evolutionary conserved domains: CADD^50^, DANN^51^ and PROVEAN^52^. Novel variants were compared across multiple specie in Ensembl^53^, to verify conservation. To determine if our gene lists were enriched in any functional categories or metabolic pathways, we performed analyses using DAVID^54^, Reactome^55^ and KEGG^56^. Finally, Enrichment map app^57^ was used to visualize the enriched gene-set as a network in Cytoscape3 platform^58^. We used simulations with other families (without AATD), to assess the power of this approach under different levels of genetic heterogeneity (data not shown).

## Supplementary information

Supplementary file 1
