# Supplementary material for: Exome Sequencing Reveals Immune Genes as Susceptibility Modifiers in Individuals with α1-Antitrypsin Deficiency
Source: Sci Rep. 2019 Sep 11;9:13088. doi: 10.1038/s41598-019-49409-1 (PMC6739380; doi:10.1038/s41598-019-49409-1)
Supplement: Supplementary file 1 [file 41598_2019_49409_MOESM1_ESM.doc]

**SUPPLEMENTARY INFORMATION**

**exome sequencing REVEALS IMMUNE GENES AS susceptibility MODIFIERS in INDIVIDUALS WITH α_1_-ANTITRYPSIN DEFICIENCY**

**Chiara Rigobello ^1#^, Simonetta Baraldo ^1#^, Mariaenrica Tinè ^1^, Ilaria Ferrarotti ^2^, Angelo Guido Corsico ^2,3^, Erica Bazzan ^1^, Graziella Turato ^1^, Elisabetta Balestro^1^, Davide Biondini ^1^, Giorgio Valle ^4^, Marina Saetta ^1*§^, Manuel G. Cosio ^1,5§^**

^1^Department of Cardiac, Thoracic, Vascular Sciences and Public Health, University of Padova, Italy, ^2^Center for Diagnosis of Inherited Alpha 1-antitrypsin Deficiency, Department of Internal Medicine and Therapeutics, University of Pavia, Italy, ^3^Division of Respiratory Diseases, IRCCS Policlinico San Matteo Foundation, Italy, ^4^CRIBI Biotechnology Center, University of Padova, Italy, ^5^Meakins-Christie Laboratories, Respiratory Division, McGill University, Montreal, Canada.

**# These authors contributed equally as first authors**

**§ These authors contributed equally as last authors**

***Corresponding author: marina.saetta@unipd.it**

**methods**

Briefly, the study included 9 individuals from 4 different families in the Italian Registry for AATD ^1^. The probands in this registry were aged ≥ 18 years with the presence of severe AAT deficiency, defined by the carriage of the PI*ZZ genotype, or null genotypic variants. Within the same family, we compared siblings who were concordant for genotype, but discordant for clinical presentation: i.e., at least one individual had to have emphysema, while the other was non-affected. To confirm that non-affected subjects were free of any respiratory disease we also required them to have a preserved lung function (FEV1/FVC > 70%), normal diffusing capacity for carbon monoxide (DL_CO_) and normal blood gases. To minimize the potential confounding effect of smoking, we have selected among the families in the Italian registry those whose smoking history was unremarkable (either nonsmokers or former smokers who had quit by >10 years and had <20 pack-years cumulative exposure). Blood samples were obtained from the patients under written consent, according to the Institution’s ethical board. All members gave consent for genetic studies. The clinical results are presented here in a fully anonymous form.

DNA was extracted from whole blood and genotyping performed as previously described ^2^. Exome libraries were prepared using the Ion Proton Targeted Sequencing Library (Ion AmpliSeq™ Exome RDY Kit) with a minimum coverage of 80X; sequencing was performed with Ion Proton™ Hi-Q™ Next-Generation sequencer. Raw sequence reads were mapped to the Genome Reference Consortium Human Build 37 (GRCh37) ^3^ using the Torrent Suite 4.0 pipeline (Life Technologies, Carlsbad, CA, USA). After removal of PCR duplicates, uniquely mapped reads were used for the analysis. The Coverage Analysis Plugin was used to provide statistics describing the level of sequence coverage produced, while the Torrent Variant Caller Plugin (TVC) was used to call single nucleotide polymorphism (SNP) and insertion/deletion (InDel) variants within the genomic regions. TVC's default parameters were applied according to attributes for Germ-Line - High Stringency variants discovery. Variants were reported in Variant Call Format (VCF) file format.

To filter and prioritize the identified variants, we used QueryOR ^4^, a new online pipeline developed by the bioinformatics unit of our university. We included only high confidence variants with: 1) coverage level >40X and 2) alternative allele coverage >30X; 3) only protein-altering substitution, including: truncating variants (stop gain/loss, start loss, or frameshift), missense variants, canonical splice site variants, InDels affecting protein-coding regions and variants within the intron-exon boundary; 4) no mAiRs (minor Allele in Reference), considered as false positive variants in the reference database. We did not apply any further filtering criteria, considering both novel and annotated variants.

We analyzed affected and non-affected subjects considering different inheritance models: a) recessive model (*shared homozygosity* in QueryOR); b) dominant model (*shared variants* in QueryOR). The analysis was restricted to variants confirmed at least in three families out of four.

ANNOVAR ^5^ and dbNSFP database (v.2.9) ^6^ were used to integrate annotations, linking variants to genes, transcripts, proteins and biological ontologies. To further estimate the impact of variants on protein structure and function, we used scores that consider the functional impact on evolutionary conserved domains: CADD ^7^, DANN ^8^ and PROVEAN ^9^. Novel variants were compared across multiple species in Ensembl ^10^, to verify conservation. To determine if our gene lists were enriched in any functional categories or metabolic pathways, we performed analyses using DAVID ^11^, Reactome ^12^ and KEGG ^13^. Finally, Enrichment map app ^14^ was used to visualize the enriched gene-set as a network in Cytoscape3 platform ^15^. We used simulations with other families (without AATD), to assess the power of this approach under different levels of genetic heterogeneity (data not shown).

**RESULTs**

WES generated an average of 3.6 x 10^7^ reads mapped to the reference genome at a mean depth of 105.4-fold coverage per sample. Any base call with Phred Quality Score <20 (Q20) was considered low quality and discarded. The 93.5% of exome was covered at minimum with 20X, and a uniformity > 91% (Supplementary Figure S1; Supplementary Table S1). The transition/transversion (Ti/Tv) ratio estimated for each sample is ≥ 2.7 (Supplementary Table S2). All these parameters exceeded the normal standards applied for the identification of germline variants, making our results robust.


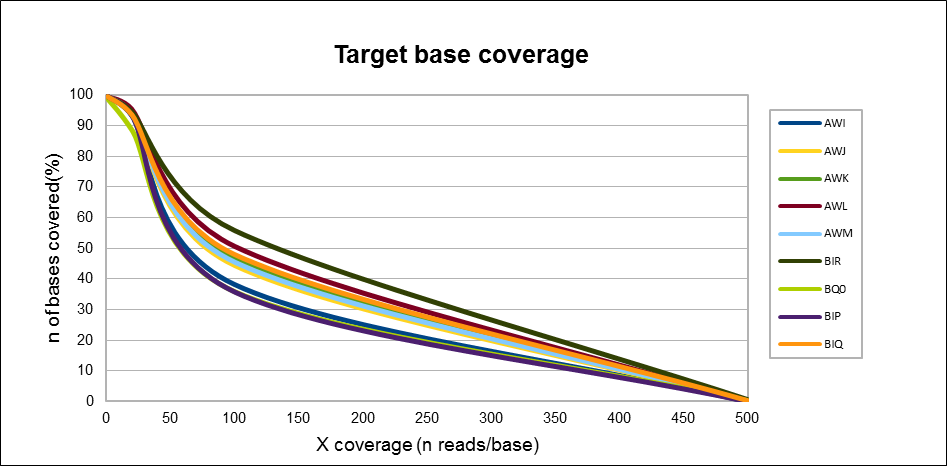
**FIGURES**

**Supplementary Figure S1: Cumulative distribution of the coverage depth.** Cumulative exome coverage curve is generated by plotting the percentage of the exome represented at different read depths, where read depth is defined as the number of individual 150-bp sequenced reads mapped to a particular exome position. Each line represents a sample; all samples have more than 50% of the exome covered at a depth between 20X–50X; the 93.5% of exome was covered with at least 20X on average.

**TableS**

**Supplementary Table S1**: Exome Sequencing Results using the Ion AmpliSeq™ Exome RDY Kit

| **Sample**  **ID** | **Total Mapped**  **Reads** | **Reads on target**  **[%]** | **Mapped bases**  **[Gb]** | **Average coverage**  **[X]** | **Uniformity**  **[%]** |
| --- | --- | --- | --- | --- | --- |
| AWI | 30960190 | 95.87 | 5.6 | 92.17 | 93.79 |
| AWJ | 35097125 | 95.78 | 6.2 | 102.2 | 94.02 |
| AWK | 35919499 | 93.71 | 6.5 | 107.4 | 93.68 |
| AWL | 39325449 | 93.67 | 6.9 | 111.7 | 94.54 |
| AWM | 37021122 | 93.42 | 6.5 | 105.2 | 93.58 |
| BIR | 44369894 | 96.46 | 7.8 | 130.3 | 92.39 |
| BQ0 | 31710717 | 96.39 | 5.7 | 93.7 | 91.39 |
| BIP | 29967132 | 96.08 | 5.4 | 89.17 | 94.20 |
| BIQ | 39052323 | 95.75 | 7.2 | 117 | 91.91 |

Statistics of reads and alignment to reference genome for all samples’s exome sequencing. The uniformity is calculated as the variance in sequencing depth across the Exome. Gb = Gigabases

**Supplementary Table S2**: Variant statistics of exome capturing

| **Sample ID** | **Total variants** | **SNPs** | **Insertion** | **Deletion** | **Sense** | **Missense** | **Nonsense** | **Frameshift** | **Ti/Tv** |
| --- | --- | --- | --- | --- | --- | --- | --- | --- | --- |
| AWI | 37282 | 35914 | 642 | 726 | 10299 | 9183 | 81 | 174 | 2.70 |
| AWJ | 37475 | 36119 | 633 | 723 | 10420 | 9282 | 90 | 185 | 2.72 |
| AWK | 37161 | 35785 | 664 | 712 | 10271 | 9152 | 82 | 165 | 2.71 |
| AWL | 38073 | 36715 | 606 | 752 | 10554 | 9335 | 91 | 174 | 2.72 |
| AWM | 37757 | 36411 | 636 | 710 | 10468 | 9274 | 88 | 166 | 2.72 |
| BIR | 37797 | 36305 | 680 | 812 | 10418 | 9268 | 74 | 154 | 2.73 |
| BQ0 | 36331 | 34934 | 630 | 767 | 9988 | 8871 | 86 | 170 | 2.74 |
| BIP | 37557 | 36072 | 684 | 801 | 10387 | 9102 | 91 | 182 | 2.74 |
| BIQ | 38212 | 36669 | 704 | 839 | 10550 | 9278 | 99 | 175 | 2.71 |

SNPs: single nucleotide polymorphisms; Ti/Tv= transition transvertion ratio

**Supplementary Table S3:** Recessive inheritance model for affected subjects. Annotation scores and GMAF description.

| Gene Symbol | SNP ID | Substitution Type | DANN | CADD | PROVEAN | GMAF |
| --- | --- | --- | --- | --- | --- | --- |
| AKNA | rs3748176 | MISSENSE | 0.63 | 5.48 | -1.17 | 0.38 |
| DNTTIP2 | rs3747965 | MISSENSE | **0.99** | 6.01 | -0.95 | 0.34 |
| FRMD1 | rs1548349 | MISSENSE | 0.37 | 0.33 | 0.08 | 0.13 |
| HJURP | rs3732215 | MISSENSE | 0.65 | 0.93 | -0.68 | - |
| HJURP | rs3806589 | MISSENSE | 0.23 | 5.70 | 0.71 | - |
| IFIH1 | rs3747517 | MISSENSE | 0.55 | **19.05** | 4.18 | 0.41 |
| KLHL3 | rs2905608 | MISSENSE | 0.74 | 0.34 | **-5.52** | 0.17 |
| KNG1 | rs710446 | MISSENSE | 0.54 | **14.24** | 1.68 | 0.42 |
| MIB2 | rs7418389 | MISSENSE | 0.72 | 8.40 | -0.10 | 0.48 |
| MS4A14 | rs3217518 | FRAMESHIFT | - | **24.40** | - | 0.41 |
| RTP2 | rs11707167 | MISSENSE | 0.44 | 2.54 | -1.25 | 0.50 |
| SLC22A16 | rs714368 | MISSENSE | 0.19 | <0.01 | 0.01 | 0.31 |
| THSD7B | rs10206850 | MISSENSE | **0.91** | **22.60** | 0.44 | 0.41 |
| TMPRSS5 | rs7110736 | MISSENSE | 0.84 | **17.20** | 2.25 | 0.23 |
| TSPAN8 | rs3763978 | MISSENSE | **1.00** | **24.10** | **-5.91** | 0.23 |

For each variant substitution type, DANN, CADD and PROVEAN scores are shown. The last column indicates the GMAF, according to 1000 Genome Project. Bold characters show scores equal or above a defined threshold, predicted to have a deleterious effect for the protein (DANN ≥ 0.9; CADD ≥ 10; PROVEAN ≤ -2.50).

Supplementary Table S4: Recessive inheritance model for not-affected subjects. Annotation scores and GMAF description.

| Gene Symbol | SNP ID | Substitution Type | DANN | CADD | PROVEAN | GMAF |
| --- | --- | --- | --- | --- | --- | --- |
| AAK1 | rs66931661 | INFRAME | - | **12.89** | - | 0.36 |
| ABCB11 | rs2287622 | MISSENSE | **0.97** | **19.22** | 0.01 | 0.41 |
| ALG1L | rs3828357 | MISSENSE | **0.96** | 8.19 | **-2.91** | 0.28 |
| C1orf227 | rs10864004 | MISSENSE | 0.07 | < 0.01 | 2.08 | 0.34 |
| CD5 | rs2229177 | MISSENSE | **1.00** | **20.20** | **-2.53** | 0.31 |
| CPB2 | rs1926447 | MISSENSE | 0.35 | 1.99 | -0.16 | 0.22 |
| CSNK1A1L | rs9576175 | MISSENSE | 0.25 | 2.99 | 2.33 | 0.40 |
| HLA-C | rs79636386 | MISSENSE | 0.51 | 0.03 | -0.39 | 0.37 |
| HLA-DQB1 | rs1049130 | MISSENSE | 0.34 | - | - | 0.33 |
| KRTAP19-4 | rs2298437 | MISSENSE | 0.72 | 5.66 | 2.67 | 0.39 |
| LCE5A | rs2105117 | MISSENSE | 0.85 | **12.16** | **-4.79** | 0.48 |
| MMRN2 | rs3750823 | MISSENSE | 0.78 | 7.72 | -0.25 | 0.42 |
| NFATC4 | rs7149586 | MISSENSE | **0.98** | **15.39** | 0.18 | 0.36 |
| PM20D1 | rs1361754 | MISSENSE | 0.50 | 6.94 | -0.30 | 0.28 |
| RTP1 | rs6764714 | MISSENSE | 0.62 | 9.61 | -0.48 | 0.43 |
| SIM2 | rs2073601 | MISSENSE | **1.00** | **25.00** | -0.14 | 0.17 |
| TINAG | rs1058768 | MISSENSE | 0.26 | **16.84** | 3.54 | 0.26 |
| TMED5 | rs1060622 | MISSENSE | **1.00** | **23.70** | **-3.11** | 0.49 |
| TMEM173 | rs1131769 | MISSENSE | 0.22 | 9.89 | 4.47 | 0.13 |
| TPTE | Novel variant | MISSENSE | 0.52 | < 0.01 | -0.98 | - |
| ZC3H13 | rs9534264 | MISSENSE | 0.21 | 0.03 | 0.56 | 0.22 |

For each variant substitution type, DANN, CADD and PROVEAN scores are shown. The last column indicates the GMAF, according to 1000 Genome Project. Bold characters show scores equal or above a defined threshold, predicted to have a deleterious effect for the protein (DANN ≥ 0.9; CADD ≥ 10; PROVEAN ≤ -2.50).

**Supplementary Table S5:** Dominant inheritance model for affected subjects. Annotation scores and GMAF description.

| Gene name | SNP ID | Substitution Type | DANN | CADD | PROVEAN | GMAF |
| --- | --- | --- | --- | --- | --- | --- |
| ACBD3 | Novel variant | FRAMESHIFT | - | - | - | - |
| ALAD | rs1800435 | MISSENSE | 0.27 | **19.86** | 4.88 | 0.06 |
| APEX1 | rs1130409 | MISSENSE | 0.66 | **13.44** | -0.12 | 0.38 |
| EGFL8 | rs3096697 | MISSENSE | **0.91** | **15.18** | -0.17 | 0.14 |
| HSD17B4 | rs25640 | MISSENSE | **1.00** | **34.00** | **-3.39** | 0.36 |
| IQCG | rs9880989 | MISSENSE | 0.26 | < 0.01 | -0.59 | 0.28 |
| KLC2 | rs2276036 | MISSENSE | 0.87 | **16.00** | -0.20 | 0.19 |
| L3MBTL4 | rs3737353 | MISSENSE | 0.51 | 4.91 | -0.24 | 0.38 |
| LIPK | rs1214464 | MISSENSE | **0.98** | **22.20** | **-2.67** | 0.19 |
| LRCH4 | Novel variant | FRAMESHIFT | - | - | - | - |
| MKI67 | rs11106 | MISSENSE | 0.14 | < 0.01 | 0.96 | 0.42 |
| MKI67 | rs8473 | MISSENSE | 0.12 | - | 1.09 | 0.46 |
| MS4A12 | rs2298553 | NONSENSE | **1.00** | **33.00** | - | 0.48 |
| PCDH12 | rs164515 | MISSENSE | 0.70 | 2.01 | -0.26 | 0.15 |
| PIK3AP1 | rs17112076 | MISSENSE | **0.95** | 5.79 | -1.95 | 0.13 |
| PPT2 | rs116326274 (EVS)* | MISSENSE | - | - | -0.16 | 0.16 |
| PRDM16 | rs2493292 | MISSENSE | **1.00** | **23.40** | -0.23 | 0.11 |
| TBC1D26 | rs11650318 | MISSENSE | **1.00** | - | - | 0.25 |
| TBC1D26 | rs17855672 | MISSENSE | 0.80 | - | - | 0.25 |
| TRIM16 | rs1060903 | MISSENSE | 0.73 | 8.26 | - | 0.24 |
| TSPYL1 | rs3828743 | MISSENSE | **0.98** | 8.47 | -2.28 | 0.35 |
| VSTM4 | rs13088 | MISSENSE | 0.22 | 4.44 | 3.14 | 0.50 |
| ZNF286A | rs3760299 | MISSENSE | **0.99** | 9.57 | -1.34 | 0.49 |

For each variant substitution type, DANN, CADD and PROVEAN scores are shown. The last column indicates the GMAF, according to 1000 Genome Project. Bold characters show scores equal or above a defined threshold, predicted to have a deleterious effect for the protein (DANN ≥ 0.9; CADD ≥ 10; PROVEAN ≤ -2.50).

(EVS)* = present in Exome Variant Server ^16^

**Supplementary Table S6:** Dominant inheritance model for not-affected subjects. Annotation scores and GMAF description.

| Gene name | SNP ID | Substitution Type | DANN | CADD | PROVEAN | GMAF |
| --- | --- | --- | --- | --- | --- | --- |
| ACBD3 | rs2306120 | MISSENSE | **0.99** | **12.84** | -1.35 | 0.47 |
| ALPK2 | rs9944810 | MISSENSE | 0.28 | 7.63 | 1.64 | 0.48 |
| ANKK1 | rs1800497 | MISSENSE | 0.17 | 7.42 | 0.86 | 0.33 |
| ART4 | rs11276 | MISSENSE | 0.54 | **13.04** | 2.09 | 0.29 |
| BBS12 | rs309370 | MISSENSE | 0.77 | **11.94** | 0.13 | 0.49 |
| BBS12 | rs13135778 | MISSENSE | 0.24 | 3.91 | 0.95 | 0.09 |
| C12orf60 | rs7307438 | MISSENSE | 0.80 | 9.88 | **-3.61** | 0.19 |
| C12orf60 | rs139293175 | FRAMESHIFT | - | **23.10** | - | 0.19 |
| CCDC144NL | rs79930314 | NONSENSE | 0.74 | 0.91 | - | - |
| CCDC144NL | rs79843086 | MISSENSE | 0.36 | 1.06 | 0.27 | - |
| CCHCR1 | rs130068 | MISSENSE | 0.86 | **13.87** | -0.73 | 0.41 |
| CD200 | rs2272022 | MISSENSE | 0.70 | **17.87** | -1.86 | 0.20 |
| CWF19L2 | rs659040 | MISSENSE | 0.21 | < 0.01 | -0.18 | 0.11 |
| CYP21A2 | rs397515530 | MISSENSE | 0.63 | 3.29 | -0.38 | 0.21 |
| DHRS4 | rs17099455 | MISSENSE | **0.96** | **10.41** | -0.85 | 0.20 |
| DYNC2LI1 | rs9309107 | MISSENSE | 0.62 | 4.68 | -0.07 | 0.45 |
| EDN1 | rs5370 | MISSENSE | **0.97** | 6.65 | -0.89 | 0.25 |
| EFS | rs2231798 | MISSENSE | 0.81 | **21.50** | -0.25 | 0.46 |
| EPCAM | rs1126497 | MISSENSE | 0.74 | **16.30** | 2.21 | 0.33 |
| FHAD1 | rs4661330 | MISSENSE | **1.00** | **22.10** | **-4.04** | 0.25 |
| HINFP | rs100803 | MISSENSE | 0.61 | 6.90 | -0.21 | 0.40 |
| HLA-C | rs79636386 | MISSENSE | 0.51 | 0.03 | 0.42 | 0.37 |
| HLA-DQB1 | rs1130398 | MISSENSE | 0.82 | **14.95** | -2.07 | 0.40 |
| HLA-DQB1 | rs1063323 | MISSENSE | 0.76 | 2.33 | -2.13 | 0.40 |
| HLA-DRB1 | rs71547382 | MISSENSE | **0.97** | **22.10** | -1.44 | - |
| KIF20B | rs1886997 | MISSENSE | 0.13 | < 0.01 | 0.44 | 0.36 |
| KIF20B | rs144593231 | INFRAME | - | 0.61 | - | 0.36 |
| KLHL33 | rs1953225 | MISSENSE | **0.98** | **22.10** | **-2.59** | 0.26 |
| KRT40 | rs9908304 | MISSENSE | **1.00** | **24.10** | **-4.13** | 0.34 |
| LRRC6 | rs2293979 | MISSENSE | 0.53 | < 0.01 | 0.02 | 0.49 |
| MCCD1 | rs2259435 | MISSENSE | **0.90** | 3.93 | -1.80 | 0.17 |
| MICB | rs1051788 (EVS)* | MISSENSE | 0.86 | 2.50 | **-2.82** | - |
| MICB | rs1065075 (EVS)* | MISSENSE | 0.09 | < 0.01 | 2.20 | - |
| MMP27 | rs1276286 | MISSENSE | 0.34 | 0.16 | -0.77 | 0.44 |
| NOS2 | rs2297518 | MISSENSE | **1.00** | **25.70** | **-2.79** | 0.17 |
| NPAS2 | rs9223 | MISSENSE | **0.93** | **11.84** | -0.40 | 0.25 |
| NPY4R | rs79871698 | MISSENSE | **0.99** | 3.15 | -1.61 | 0.12 |
| NRG3 | rs1884282 | MISSENSE | 0.62 | 5.54 | -0.23 | 0.14 |
| NSUN4 | rs3737744 | MISSENSE | **0.99** | **21.70** | -1.39 | 0.30 |
| NUMBL | rs749669311 | INFRAME | - | - | - | - |
| OR10G2 | rs41314525 | MISSENSE | **0.99** | **19.83** | **-4.08** | 0.23 |
| OR13C2 | rs10156474 | MISSENSE | **1.00** | **24.90** | -3.12 | 0.43 |
| OR13C2 | rs10991326 | MISSENSE | **0.98** | **22.70** | **-5.10** | 0.37 |
| OR13C5 | rs4117966 | MISSENSE | **0.99** | **25.20** | **-10.90** | 0.36 |
| OR13C5 | rs1523678 | MISSENSE | 0.54 | < 0.01 | -0.76 | 0.44 |
| OR13C5 | rs1851725 | MISSENSE | 0.39 | 8.50 | **-5.43** | 0.44 |
| OR13C5 | rs6479260 | MISSENSE | 0.19 | < 0.01 | 3.07 | 0.42 |
| OR13C5 | rs11314210 | FRAMESHIFT | - | **23.60** | - | 0.44 |
| OR13C9 | rs993658 | MISSENSE | **0.95** | **15.23** | -1.02 | 0.35 |
| OR2T2 | rs67700848 | MISSENSE | 0.11 | < 0.01 | -0.15 | - |
| OR4E2 | rs61732411 | MISSENSE | **1.00** | **15.79** | **-2.79** | 0.14 |
| PRDM1 | rs811925 | MISSENSE | 0.83 | **22.30** | 0.92 | 0.15 |
| PSMD9 | rs14259 | MISSENSE | **1.00** | **16.57** | **-3.54** | 0.29 |
| RNLS | rs2296545 | MISSENSE | **0.99** | **13.12** | -1.22 | 0.48 |
| SPATA16 | rs1515441 | MISSENSE | **1.00** | **23.80** | -1.56 | 0.17 |
| SPATA16 | rs16846616 | MISSENSE | **0.99** | **23.30** | **-3.46** | 0.14 |
| SVEP1 | rs3739451 | MISSENSE | **0.91** | **15.98** | -0.53 | 0.11 |
| TMEM132C | rs12424159 | MISSENSE | 0.58 | 4.27 | -1.14 | 0.49 |
| TMEM71 | rs1895807 | MISSENSE | 0.36 | 0.72 | 0.21 | 0.42 |
| WDR66 | rs17852561 | MISSENSE | **0.99** | **15.49** | -1.38 | 0.28 |
| WWC2 | rs11734376 | MISSENSE | 0.81 | **15.83** | -0.81 | 0.06 |
| ZNF626 | rs73002662 | MISSENSE | **0.98** | **11.04** | - | 0.09 |

For each variant substitution type, DANN, CADD and PROVEAN scores are shown. The last column indicates the GMAF, according to 1000 Genome Project. Bold characters show scores equal or above a defined threshold, predicted to have a deleterious effect for the protein (DANN ≥ 0.9; CADD ≥ 10; PROVEAN ≤ -2.50).

(EVS)* = present in Exome Variant Server ^16^

**Supplementary Table S7**: Reactome Enrichment for the recessive inheritance model in affected subjects.

| **Pathway name** | **Found** | **Total** | **p-value** | **FDR** |
| --- | --- | --- | --- | --- |
| NF-kB activation through FADD/RIP-1 pathway mediated  by caspase-8 and -10 | 1 | 12 | 1.52E-2 | 1.28E-1 |
| TRAF3-dependent IRF activation pathway | 1 | 14 | 1.77E-2 | 1.28E-1 |

**Supplementary Table S8**: Reactome Enrichment for the recessive inheritance model in not-affected subjects.

| **Pathway name** | **Found** | **Total** | **p-value** | **FDR** |
| --- | --- | --- | --- | --- |
| Antigen Presentation: Folding. assembly and peptide loading of class I MHC | 14 | 93 | 1.11E-16 | **1.11E-15** |
| Endosomal/Vacuolar pathway | 14 | 79 | 1.11E-16 | **1.11E-15** |
| ER-Phagosome pathway | 14 | 152 | 1.11E-16 | **1.11E-15** |
| Antigen processing-Cross presentation | 14 | 168 | 1.11E-16 | **1.11E-15** |
| Interferon gamma signaling | 15 | 172 | 1.11E-16 | **1.11E-15** |
| Interferon alpha/beta signaling | 14 | 137 | 1.11E-16 | **1.11E-15** |
| Interferon Signaling | 15 | 284 | 3.11E-15 | **2.49E-14** |
| Immunoregulatory interactions between a Lymphoid and a non-Lymphoid cell | 14 | 297 | 1.53E-13 | **1.07E-12** |
| Class I MHC mediated antigen processing & presentation | 14 | 440 | 2.89E-11 | **1.73E-10** |
| Cytokine Signaling in Immune system | 15 | 1.013 | 1.61E-7 | **9.66E-7** |
| Adaptive Immune System | 15 | 1.108 | 5.17E-7 | **2.59E-6** |
| Immune System | 17 | 2.444 | 5.48E-4 | **2.74E-3** |
| Synthesis of PIPs at the Golgi membrane | 2 | 18 | 1.54E-3 | **6.18E-3** |
| Defective ABCB11 causes progressive familial intrahepatic cholestasis 2 and benign recurrent intrahepatic cholestasis 2 | 1 | 1 | 3.18E-3 | **1.27E-2** |
| STAT6-mediated induction of chemokines | 1 | 3 | 9.52E-3 | **3.81E-2** |
| PI Metabolism | 2 | 84 | 2.95E-2 | 8.85E-2 |
| IRF3-mediated induction of type I IFN | 1 | 13 | 4.06E-2 | 1.06E-1 |
| Regulation of innate immune responses to cytosolic DNA | 1 | 15 | 4.67E-2 | 1.06E-1 |
| STING mediated induction of host immune responses | 1 | 16 | 4.98E-2 | 1.06E-1 |
| Recycling of bile acids and salts | 1 | 16 | 4.98E-2 | 1.06E-1 |
| Metabolism of Angiotensinogen to Angiotensins | 1 | 17 | 5.28E-2 | 1.06E-1 |
| Synthesis of bile acids and bile salts via 7alpha-hydroxycholesterol | 1 | 24 | 7.38E-2 | 1.48E-1 |
| WNT ligand biogenesis and trafficking | 1 | 26 | 7.97E-2 | 1.53E-1 |

Column “Found” shows the number of molecules in the data set found in the pathway.

“Total” column shows the total number of molecules within the pathway. p-value is set to ≤ 0.05.

The last column shows the False Discovery Rate (FDR) corrected p-value.

**Supplementary Table S9** Reactome Enrichment for the dominant inheritance model in affected subjects.

| **Pathway name** | **Found** | **Tot**al | **p-value** | **FDR** |
| --- | --- | --- | --- | --- |
| RHO GTPases activate KTN1 | 2 | 12 | 1.97E-04 | 2.21E-02 |
| Abasic sugar-phosphate removal via the single-nucleotide replacement pathway | 1 | 3 | 5.09E-03 | 1.85E-01 |
| Kinesins | 2 | 67 | 5.80E-03 | 1.85E-01 |
| Resolution of AP sites via the single-nucleotide replacement pathway | 1 | 7 | 1.18E-02 | 1.85E-01 |
| COPI-dependent Golgi-to-ER retrograde traffic | 2 | 106 | 1.40E-02 | 1.85E-01 |
| Displacement of DNA glycosylase by APEX1 | 1 | 9 | 1.52E-02 | 1.85E-01 |
| POLB-Dependent Long Patch Base Excision Repair | 1 | 15 | 2.52E-02 | 1.85E-01 |
| Golgi-to-ER retrograde transport | 2 | 147 | 2.57E-02 | 1.85E-01 |
| MHC class II antigen presentation | 2 | 147 | 2.57E-02 | 1.85E-01 |
| Factors involved in megakaryocyte development and platelet production | 2 | 179 | 3.69E-02 | 1.85E-01 |
| PCNA-Dependent Long Patch Base Excision Repair | 1 | 23 | 3.84E-02 | 1.85E-01 |

**Supplementary Table S10**: Reactome Enrichment for the dominant inheritance model in not-affected subjects.

| **Pathway name** | **Found** | **Total** | **p-value** | **FDR** |
| --- | --- | --- | --- | --- |
| Translocation of ZAP-70 to Immunological synapse | 16 | 42 | 1.11E-16 | **3.77E-15** |
| MHC class II antigen presentation | 18 | 147 | 1.11E-16 | **3.77E-15** |
| Phosphorylation of CD3 and TCR zeta chains | 16 | 45 | 1.11E-16 | **3.77E-15** |
| TCR signaling | 17 | 146 | 1.11E-16 | **3.77E-15** |
| PD-1 signaling | 16 | 45 | 1.11E-16 | **3.77E-15** |
| Adaptive Immune System | 36 | 1.189 | 1.11E-16 | **3.77E-15** |
| Generation of second messenger molecules | 16 | 58 | 1.11E-16 | **3.77E-15** |
| Downstream TCR signaling | 17 | 124 | 1.11E-16 | **3.77E-15** |
| Interferon gamma signaling | 30 | 176 | 1.11E-16 | **3.77E-15** |
| Interferon Signaling | 30 | 292 | 1.11E-16 | **3.77E-15** |
| Costimulation by the CD28 family | 16 | 98 | 1.11E-16 | **3.77E-15** |
| Endosomal/Vacuolar pathway | 14 | 82 | 2.22E-16 | **6.88E-15** |
| Antigen Presentation: Folding. assembly and peptide loading of class I MHC | 14 | 102 | 3.77E-15 | **1.09E-13** |
| Cytokine Signaling in Immune system | 34 | 1.222 | 1.23E-14 | **3.33E-13** |
| ER-Phagosome pathway | 15 | 164 | 1.23E-13 | **3.08E-12** |
| Interferon alpha/beta signaling | 14 | 141 | 2.91E-13 | **6.69E-12** |
| Antigen processing-Cross presentation | 15 | 186 | 7.31E-13 | **1.61E-11** |
| Immunoregulatory interactions between a Lymphoid and a non-Lymphoid cell | 16 | 316 | 1.13E-10 | **2.37E-9** |
| Class I MHC mediated antigen processing & presentation | 15 | 464 | 1.71E-7 | **3.41E-6** |
| Immune System | 38 | 2.765 | 2.87E-7 | **5.45E-6** |
| Regulation of TP53 Expression | 2 | 4 | 3.01E-4 | **5.41E-3** |
| Olfactory Signaling Pathway | 7 | 432 | 1.82E-2 | 3.09E-1 |
| RORA activates gene expression | 2 | 38 | 2.37E-2 | 3.79E-1 |
| Defective CYP21A2 causes Adrenal hyperplasia 3 (AH3) | 1 | 5 | 3.06E-2 | 4.59E-1 |
| Regulation of TP53 Expression and Degradation | 2 | 46 | 3.36E-2 | 4.96E-1 |

Column “Found” shows the number of molecules in the data set found in the pathway.

“Total” column shows the total number of molecules within the pathway. p-value is set to ≤ 0.05.

The last column shows the False Discovery Rate (FDR) corrected p-value.

**REFERENCES**

1. Luisetti, M. *et al.* Italian registry of patients with alpha-1 antitrypsin deficiency: general data and quality of life evaluation. *Copd* **12 Suppl 1**, 52–57 (2015).

2. Ferrarotti, I. *et al.* Serum levels and genotype distribution of alpha1-antitrypsin in the general population. *Thorax* **67**, 669–674 (2012).

3. *hg19 release 81 of human gene. Available from http://grch37.ensembl.org/info/data/ftp/.* (2015).

4. Bertoldi, L. *et al.* QueryOR: a comprehensive web platform for genetic variant analysis and prioritization. *BMC Bioinformatics* **18**, 225–017–1654–4 (2017).

5. Yang, H. & Wang, K. Genomic variant annotation and prioritization with ANNOVAR and wANNOVAR. *Nat. Protoc.* **10**, 1556–1566 (2015).

6. Liu, M., Watson, L. T. & Zhang, L. Predicting the combined effect of multiple genetic variants. *Hum. Genomics* **9**, 18–015–0040–4 (2015).

7. Kircher, M. *et al.* A general framework for estimating the relative pathogenicity of human genetic variants. *Nat. Genet.* **46**, 310–315 (2014).

8. Quang, D., Chen, Y. & Xie, X. DANN: a deep learning approach for annotating the pathogenicity of genetic variants. *Bioinforma. Oxf. Engl.* **31**, 761–763 (2015).

9. Choi, Y. & Chan, A. P. PROVEAN web server: a tool to predict the functional effect of amino acid substitutions and indels. *Bioinforma. Oxf. Engl.* **31**, 2745–2747 (2015).

10. Herrero, J. *et al.* Ensembl comparative genomics resources. *Database* **2016**, bav096–bav096 (2016).

11. Huang, D. W., Sherman, B. T. & Lempicki, R. A. Systematic and integrative analysis of large gene lists using DAVID bioinformatics resources. *Nat. Protoc.* **4**, 44 (2008).

12. Croft, D. *et al.* The Reactome pathway knowledgebase. *Nucleic Acids Res.* **42**, D472–7 (2014).

13. Kanehisa, M., Goto, S., Sato, Y., Furumichi, M. & Tanabe, M. KEGG for integration and interpretation of large-scale molecular data sets. *Nucleic Acids Res.* **40**, D109–14 (2012).

14. Merico, D., Isserlin, R., Stueker, O., Emili, A. & Bader, G. D. Enrichment Map: A Network-Based Method for Gene-Set Enrichment Visualization and Interpretation. *PLOS ONE* **5**, e13984 (2010).

15. Shannon, P. *et al.* Cytoscape: a software environment for integrated models of biomolecular interaction networks. *Genome Res.* **13**, 2498–2504 (2003).

16. *Exome Variant Server. Available from: http://evs.gs.washington.edu/EVS/.* (2016).
